# Stereotactic Body Radiotherapy for Hepatocellular Carcinoma Resulting in a Durable Relapse-Free Survival: A Case Report

**DOI:** 10.7759/cureus.841

**Published:** 2016-10-24

**Authors:** Samual Francis, Ned Williams, Christopher J Anker, Akram Shaaban, Robin Kim, Dennis Shrieve, Jonathan Tward

**Affiliations:** 1 Radiation Oncology, University of Utah Huntsman Cancer Hospital; 2 Radiation Oncology, University of Vermont; 3 Radiology, University of Utah Huntsman Cancer Hospital; 4 General Surgery, University of Utah School of Medicine

**Keywords:** hepatocellular carcinoma, stereotactic body radiotherapy, liver cancer, erythropoietic protoporphyria

## Abstract

The standard of care for localized hepatocellular carcinoma (HCC) is surgical resection. For patients who decline or who are unfit for surgery, stereotactic body radiotherapy (SBRT) is emerging as a viable treatment approach. We present a case of a 77-year-old female in whom an early stage HCC was incidentally discovered. Given her religious edicts as a devout Jehovah’s Witness and her subsequent desire to avoid a blood transfusion, she declined surgical resection or transplant due to the risk of hemorrhage. Ablative therapy was deemed inappropriate given the mass’s size and location adjacent to the inferior vena cava and diaphragm. She was treated with definitive SBRT to a total dose of 60 Gy administered in three 20 Gy fractions every other day. She had a complete response to the treatment and remains without evidence of disease after 39 months of follow-up. Her only treatment-related side effect is a persistent CTCAE Grade 1 myositis on her back overlying the treatment area. We report this case to add to the growing body of literature suggesting SBRT as an effective and safe alternative treatment modality for HCC.

## Introduction

Hepatocellular carcinoma (HCC) is the sixth most prevalent cancer and the second most common cause of cancer mortality worldwide [1]. Globally, the most common risk factors for the development of HCC are chronic Hepatitis B and Hepatitis C, infection, and alcoholic cirrhosis [2-3]. Other risk factors for HCC include hemochromatosis, alpha-l-antitrypsin deficiency, glycogen storage disease, various porphyrias, tyrosinemia, and Wilson disease [3-4]. The standard of care for localized HCC is surgical resection. We present a case of a 77-year-old female in whom an early stage HCC was incidentally discovered and successfully treated with stereotactic body radiotherapy (SBRT).

## Case presentation

An asymptomatic 77-year-old Caucasian woman with a history of mild erythropoietic protoporphyria (EPP), seizure disorder, and osteoporosis was incidentally found to have an area of abnormal uptake in the liver on a bone scan. Subsequent computed tomography (CT) of the abdomen and pelvis revealed a 4.9 x 3.9 cm heterogeneous arterially enhancing mass within segment 7 of the right hepatic lobe. Magnetic resonance imaging (MRI) of the abdomen demonstrated an arterially enhancing mass with capsular enhancement and delayed washout. Areas of signal loss on the opposed-phase images compared to in-phase images were present and consistent with the presence of microscopic tumoral fat. The appearance was consistent with HCC [5-7]. Colonoscopy and esophagogastroduodenoscopy demonstrated internal hemorrhoids and mild gastritis but were otherwise normal. The patient agreed to participate and was explained the nature and objectives of this study, and informed consent was formally obtained. No reference to the patient's identity was made at any stage during data analysis or in the report.

Pertinent initial laboratory data included: alpha fetoprotein (AFP), 486 ng/mL (reference range, 0 to 15 ng/mL); alkaline phosphatase, 126 U/L (reference range, 38 to 120 U/L); AST, 26 U/L (reference range, 14 to 50 U/L); ALT, 20 U/L (reference range, 9 to 52 U/L); albumin, 4.3 gm/dL (reference range, 3.5 to 4.6 gm/dL); gamma glutamyl transferase (GGT), 107 U/L (reference range, 10 to 55 U/L); hepatitis C antibody titers, negative; bilirubin, <0.1 mg/dL (reference range, 0.2 to 1.3 mg/dL), cancer antigen 19-9 (CA-19-9), 21 U/mL (reference range, 0 to 37 U/mL); and cancer antigen 125 (CA-125), 49 U/mL (reference range 0 to 35 U/mL). She did not have ascites or encephalopathy and was Child-Pugh Class A.

Based on the lesion size, presence of characteristic imaging features, and elevated AFP, a diagnosis of localized HCC (Stage I, T1N0M0) was made. Given her religious edicts as a devout Jehovah’s Witness and subsequent desire to avoid a blood transfusion, our patient declined surgical resection or transplant due to the risk of hemorrhage. Ablative therapy was deemed inappropriate given the mass’s size and location adjacent to the inferior vena cava and diaphragm. After discussion of treatment options of either chemoembolization or radiotherapy, she opted for radiotherapy.

**Figure 1:**
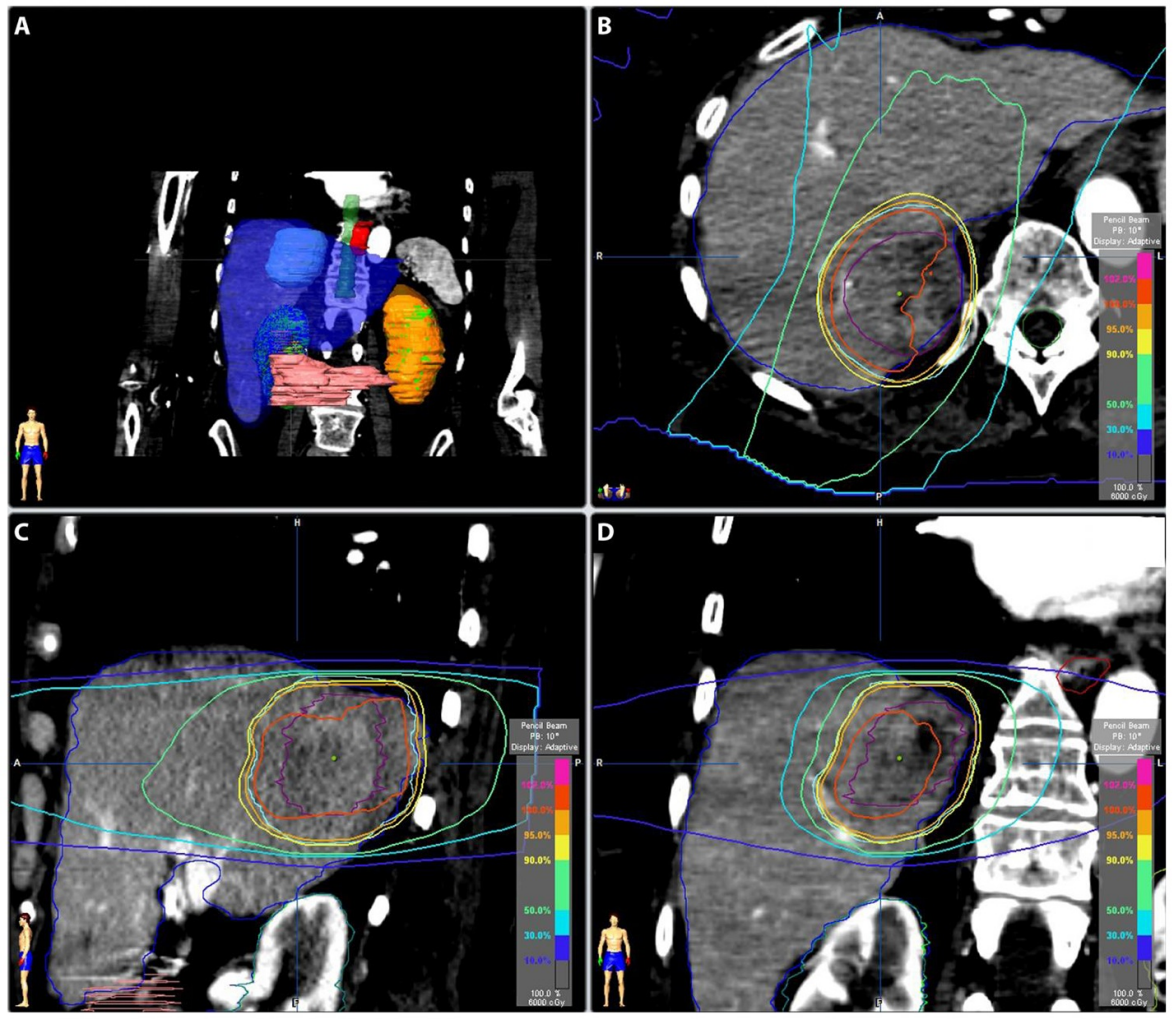
Radiation Treatment Planning Figure 1: Panel A shows the internal target volume (ITV) as a light blue mass within the liver (blue). Panels B-D show the isodose lines surrounding the ITV (purple) for axial, sagittal, and coronal views, respectively.

She was treated with definitive SBRT to a total dose of 60 Gy administered in three 20 Gy fractions every other day, prescribed to the 100% isodose line. The simulation was performed using BodyFIX® (Elekta, Atlanta, GA) immobilization with arms down and IV contrast with 4D CT. An internal target volume (ITV) was created by contouring the visible lesion on the 4D simulation CT. The ITV was expanded by 5 mm medially and superiorly to account for proximity to vertebral bodies and 10 mm in other dimensions to form the planning target volume (PTV). Her liver, ITV, and PTV volumes were 1336 cm^3^, 47 cm^3^, and 133 cm^3^, respectively. Stereotactic body radiotherapy treatment was planned using iPlan® (Brainlab, Westchester, IL). Sixty-four percent of the PTV received 100% of the prescription dose, and 98% of the PTV received 90% of the prescription dose (Figure 1).

The minimum dose to the ITV was 5852 cGy. The mean liver dose was 15.6 Gy, and 922 cm^3^ received ≤ 19.2 Gy. The max dose to the right kidney was 2.6 Gy. The spinal cord max dose was 18 Gy; 0.4 cm^3^ received 16.2 Gy, and 1.6 cm^3^ received 15 Gy. The max esophagus dose was 24.7 Gy, and 5 cm^3^ received 4.8 Gy. The chest wall of 31 cm^3^ received 30 Gy, and 9.4 cm^3^ of the skin received 30 Gy. Positional shifts were made based on a control CT scan verifying ITV location before each treatment, and she was treated using the Novalis® delivery system (Brainlab, Westchester, IL).

She reported minimal-to-no acute side effects. Over a period of months after the treatment, she developed a CTCAE Grade 1 myositis on her back overlying the treatment area. This pain has persisted to the present time and is relieved with ibuprofen.

**Figure 2:**
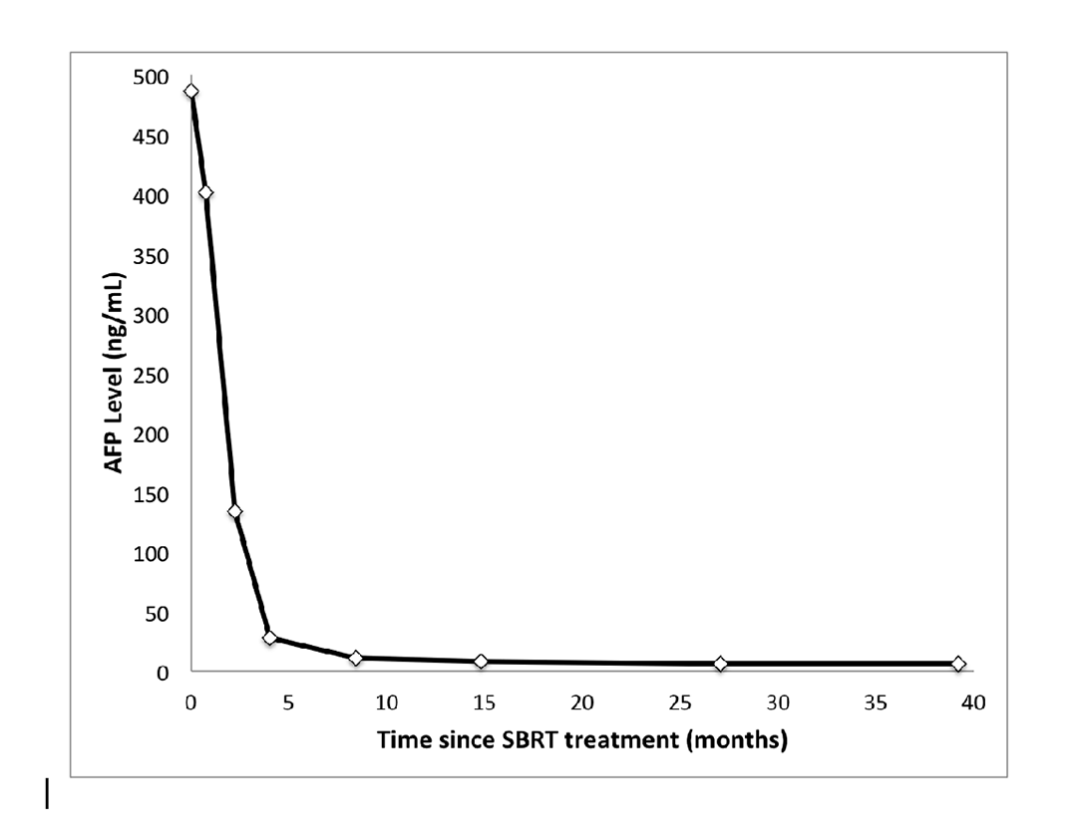
Alpha Fetoprotein Levels Figure 2: Alpha fetoprotein (AFP) level over time for this patient after stereotactic body radiotherapy (SBRT) treatment. Normal AFP reference range is 0 to 15 ng/mL. This patient’s AFP normalized eight months after SBRT.

After treatment, she was followed routinely by radiation oncology with serial magnetic resonance imaging (MRI) and serum alpha-fetoprotein (AFP) levels. Her AFP level normalized eight months after SBRT and has remained at 6 ng/mL since that time (Figure 2).

**Figure 3:**
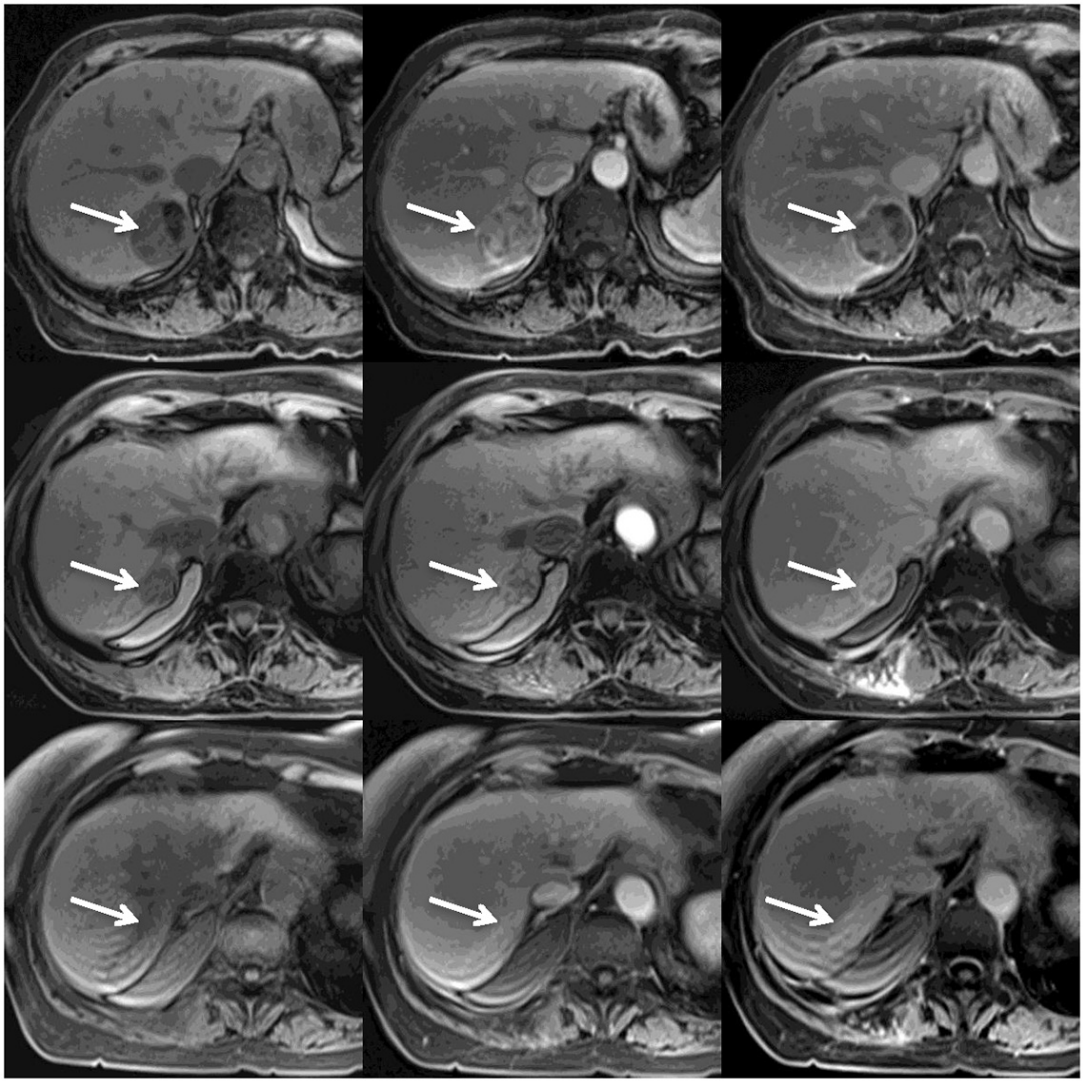
MRI Treatment Response Figure 3: Serial MRI images obtained before (upper row), 3 months after (middle row) and 39 months after (lower row) the SBRT treatment. Each row shows pre-contrast (left), arterial phase (middle) and delayed venous phase (right) MRI VIBE images. The upper row shows the classic appearance of HCC (white arrows): low signal intensity on pre-contrast image, prominent enhancement during the arterial phase, and decreased enhancement of the tumor on the delayed phase with persistent tumoral capsular enhancement. The middle row shows significant decrease in the size of the tumor, decreased enhancement during the arterial phase, and residual heterogeneous enhancement during the delayed phase. The lower row shows an ill-defined, non-mass-like area consistent with post-radiation fibrosis.

Two months after the completion of SBRT, her liver lesion decreased in size (4.3 x 4.4 cm → 3.7 x 3.3 cm), and visible shrinkage continued on subsequent imaging (Figure 3).

By 39 months posttreatment, MRI imaging showed an ill-defined area of delayed enhancement without appreciable arterial enhancement at the tumor site representing post-radiation fibrosis.

## Discussion

To our knowledge, this is the first report describing primary HCC in a patient with EPP who has no other risk factors for HCC [8]. Although the optimal management for HCC is surgical resection or transplantation, the majority of patients who present with HCC are deemed unresectable [9]. In these instances, non-surgical interventions are employed, including transarterial chemoembolization (TACE), radioembolization/external beam radiotherapy (EBRT). In the past, EBRT has been limited by the risk of radiation-induced liver disease (RILD). With the advancement of SBRT, which is the ability to deliver highly conformal, large ablative doses of radiation over a few fractions, SBRT has become an attractive option for HCC treatment. Here we present a case showing that SBRT is a curative treatment option.

Tse et al., reported results from a Phase I study of 41 patients with unresectable HCC or intrahepatic cholangiocarcinoma treated with a six-fraction SBRT regimen with a median dose of 36 Gy [10]. No RILD or toxicities ≥ Grade 4 were seen. Seven patients had a decline in liver function from Child-Pugh Class A to B within three months of SBRT. Overall, they concluded their six-fraction regimen was well-tolerated and safe [10]. Additionally, Goodman et al., reported results from a Phase I dose-escalation study of 26 patients with either primary HCC or liver metastases, all deemed unresectable, treated with single-fraction SBRT [11]. Patients with lesions ≤ 5 cm in diameter and with ≤ 5 lesions were included. They started at a dose of 18 Gy and increased in 4 Gy increments to a planned maximum dose of 30 Gy. The cumulative risk of local failure at twelve months was 23%. No toxicities ≥ Grade 3 were observed. Three Grade 2 and nine Grade 1 toxicities were reported, all gastrointestinal. More recently, Bujold et al., reported on SBRT for patients unsuitable for standard locoregional therapies for locally advanced HCC. In their report, 102 patients with HCC were treated with SBRT at a dose range of 24 to 54 Gy in six fractions. A one-year local control of 87% and median overall survival of 17 months were reported. Grade 3 or higher toxicity was reported in 30% of patients [12]. Overall, these studies show that SBRT can be delivered safely and effectively for primary HCC, with high rates of local control. Our case further illustrates that SBRT can be a curative treatment modality for HCC.

## Conclusions

We report this case to add to the growing body of data suggesting SBRT as an effective alternative treatment modality for HCC. Further evaluation is needed in the form of randomized, prospective clinical trials to better evaluate the efficacy of SBRT as an alternative curative intent treatment for HCC. Important data will undoubtedly be gained from the currently enrolling RTOG 1112, a randomized Phase III study of sorafenib versus SBRT followed by sorafenib in HCC.
